# Navigating the U.S. health insurance landscape for children with rare diseases: a qualitative study of parents’ experiences

**DOI:** 10.1186/s13023-021-01943-w

**Published:** 2021-07-15

**Authors:** Tai L. S. Pasquini, Sarah L. Goff, Jennifer M. Whitehill

**Affiliations:** 1 Department of Health Promotion and Policy, School of Public Health and Health Sciences, University of Massachusetts Amherst, 715 North Pleasant Street, Amherst, MA United States; 2 Congenital Hyperinsulinism International, P.O. Box 135, Glen Ridge, NJ 07028 USA

**Keywords:** Rare diseases, Parent, Caregiver, Health insurance, Healthcare access, Qualitative research

## Abstract

**Background:**

Parents of children with rare diseases often face uncertainty about diagnosis, treatment, and costs associated with healthcare for their child. Health insurance status impacts each of these areas, but no U.S. study has explored parents’ perceptions of the health insurance impacts on their child’s care. This study aimed to qualitatively explore how these parents navigate the complex health insurance system for their children and their experiences in doing so.

**Methods:**

Semi-structured interviews were conducted with parents of children with metachromatic leukodystrophy (MLD) and spinal muscular atrophy (SMA), chosen for specific disease characteristics and orphan drug status. Participants were recruited via e-mail through patient advocacy organizations between September and December 2018. Interviews were conducted via Skype, were recorded, and professionally transcribed. Modified grounded theory was utilized as a methodology to analyze transcripts in an iterative process to determine themes and sub-themes based on participant described experiences.

**Results:**

Major themes and subthemes that emerged across the 15 interviews included: (1) difficulties obtaining secondary insurance based on state eligibility criteria; (2) difficulty accessing needed healthcare services; and (3) need for repeated interactions with insurance representatives. The absence of clearly documented or widely recognized clinical guidelines exacerbated the difficulty accessing care identified as necessary by their healthcare team, such as therapy and equipment. An explanatory model for parent’s experiences was developed from the themes and subthemes. The model includes the cyclical nature of interacting with insurance for redundant reauthorizations and the outside support and financial assistance that is often necessary to address their child’s healthcare needs.

**Conclusions:**

With complex health conditions, small setbacks can become costly and disruptive to the health of the child and the life of the family. This study suggests that patients with rare diseases may benefit from time limits for processing coverage decisions, increasing transparency in the claims and preauthorization processes, and more expansive authorizations for on-going needs. Additional studies are needed to understand the full scope of barriers and to inform policies that can facilitate better access for families living with rare diseases.

## Background

Rare diseases are defined in the United States as conditions with less than 200,000 cases [1, 2]. The National Institutes of Health (NIH) currently lists over 6,800 rare diseases that together impact between 25 to 30 million Americans, 60% of whom are under 18 years of age [3]. Rare diseases are often difficult to diagnose, have few treatment options, and limited research on the natural history of the condition. These challenges, along with an inadequate number of healthcare providers familiar with rare diseases [4–9] and the financial strain generated by rare disease care often leave parents of children living with rare diseases feeling isolated and uncertain about the disease progression [5, 7, 10–13].

The knowledge gaps about rare diseases and related care often places parents in the role of disease expert and care coordinator [6, 14–16]. They often have to identify services across siloed healthcare and social service agencies [12, 16, 17] and may need to educate some healthcare providers, who can serve as gatekeepers to certain treatments or services [15]. To avoid large out-of-pocket bills, parents must interface with health insurance representatives to advocate for coverage of services.

Parents’ role in interfacing with insurance representatives may be the most complex aspect of their advocacy role. Like healthcare delivery systems in the U.S., the insurance system is highly complex, fragmented, and eligibility for plans varies based on sociodemographic factors, geography, and employment status [18–21]. In 2016, 49% of Americans purchased health insurance through employer sponsored plans (private), 35% were insured by government-sponsored health insurance plans (public), and 9% were uninsured [22]. The largest public insurance programs are Medicare and Medicaid. All individuals over the age of 65 and those with long-term disabilities are eligible for Medicare [23] while Medicaid is available for people with low-income levels or certain disabilities. Eligibility for Medicaid is determined at the state level and in 2020 49.5% of enrollees nationwide were children [24].

Access to healthcare for children with rare diseases has become even more complicated as health insurance companies have sought to decrease healthcare costs at the individual and systems level. Insurers’ strategies for decreasing costs include managed care plans, which require patients to pay more if they receive care outside of a specific network of healthcare providers; requiring patients to pay a greater portion of costs; and having ‘tiered’ drug formularies that make some medications far more expensive than others [25–27]. Children with a rare disease diagnosis may need numerous visits to specialists in multiple locations [8] and a condition may only have a handful of experts or specialty centers able to manage the disease, who are unlikely to be in a families’ approved network of healthcare providers [9]. Because private insurance is tied to an employer, parents of children with rare diseases often restrict their employment choices based on the health insurance a given employer offers [13, 28, 29].

A recent U.S. study estimated that direct and indirect costs related to just 379 rare diseases represented economic costs of nearly $1 trillion in 2019 [30]. Based on their disability status, individuals may qualify for multiple insurance programs, or due to high out-of-pocket costs they may choose to purchase supplemental insurance, resulting in ‘double’ and ‘triple’ coverage. Orphan drugs are one aspect of care costs for some patients. Roughly 300 rare diseases have a Food and Drug Administration (FDA) approved orphan drug [31] and there has been much criticism of the financial incentives and price for these treatments [32–35]. A study of U.S. health insurance plans showed that orphan drug coverage is inconsistent across exchange plans, plans available for anyone within that state to purchase [36]. Despite the high cost of orphan drugs, the impact of these treatments can be life-changing for families [37].

There is a complicated balance between decreasing healthcare costs and meeting patient needs. To date, there have been no published studies of parents’ perspectives on their experiences with healthcare insurance companies or studies that seek to understand how health insurance policies may impede health care for patients with rare diseases. The aim of the study was to qualitatively explore rare disease parents’ experiences with health insurance for their child living with a rare disease, to identify potential barriers to optimal care, and to generate recommendations to address barriers identified.

## Methods

### Overview

The heterogeneity of rare diseases makes it difficult to identify a “typical” disease. Parents of children with MLD and SMA were selected for inclusion in this study because of disease similarities and orphan drug status differences for the two diseases. Both diseases have an identified genetic mutation and varying degrees of severity within the disease [38, 39]. Both conditions impact motor function, which may require physical therapy or supportive equipment to aid mobility [38–41]. SMA can impact the ability to breathe and eat, which may result in the need for feeding tubes or monitoring from a pulmonologist. MLD causes progressive deterioration of intellectual functions, seizures, an inability to speak, blindness, and hearing loss requiring a range of therapists and specialists to provide on-going care [39].In 2016, the drug nusinersen was approved by the FDA to treat SMA after clinical trials showed that it can improve motor-milestone responses for some users [37, 42]. Stem cell transplantation may be appropriate for some patients with MLD, but at the time of the study, there was no approved orphan drug available [43]. Additional MLD and SMA care for most patients focuses on symptom management.

In this qualitative study, semi-structured interviews were conducted with parents of children with one of these two rare diseases, aiming to better understand how parents’ experiences related to health insurance may impact children’s care. Modified grounded theory principles were applied through data collection techniques, an iterative process of data analysis and coding, and theory generation of a model to further tell the story as described by parents [44–46].

### Sampling and recruitment

Participants were eligible to participate in this study if they were over 18 years of age, a resident of the U.S., and the medical or legal guardian of a living individual diagnosed with SMA or MLD. Recruitment messages that included a link to the study pre-participation questionnaire were provided to patient organizations, that represented each rare disease; the organizations then emailed the messages to their members (roughly 7,200 SMA and 600 MLD families) and shared the message and links to the screening questionnaire on their organizational Facebook pages between September 2018 and December 2018. The questionnaire determined eligibility, collected contact and basic demographic information, and identified options for scheduling an audio interview via Skype. Although several interviewees offered to contact people they thought might be interested in participating, no referees contacted the study staff directly. It is possible that some of the people recruited through Facebook or email had heard about the study through peers but we did not collect these data [47]. This study was approved by the University of Massachusetts Amherst Institutional Review Board.

### Data collection and analysis

Interviews were conducted by TP using a semi-structured interview guide that included questions related to interviewees’ experiences with health insurers. Interviews were audio recorded and professionally transcribed verbatim with participants’ permission. Interview transcripts were analyzed using NVivo 12 software [48]. The section headers of the interview guide, such as “cost sharing” and “navigation” served as initial broad coding categories. The first author read the first several transcripts to gain a sense of understanding of the data as a whole then proceeded with iterative line-by-line open coding of 2–3 transcripts at a time before conducting the next set of interviews. Constant comparative analysis continued as new interviews were conducted; memos were used to identify emerging themes and to begin to build theory regarding parents’ experiences [45, 46].

Recruitment ended when data saturation had been reached, meaning no new themes emerged over three consecutive transcripts [49]. A research assistant (RA) trained in qualitative methods independently performed line-by-line coding of four transcripts after interviews were completed and side-by-side comparisons were performed between the work completed by the author and the RA to assess reproducibility of the codebook. Differences were resolved through discussion and the final side-by-side comparison found less than 5% difference between the two coders.

Axial coding was performed as the analysis progressed, continuing to build theory and construct a model that demonstrated how parents experience the health insurance system and factors that contribute to their ability to both navigate the system and gain access to care [45]. The model was developed through theory generation by exploring the linkage between the key themes and sub-themes and organizing them thematically to provide context to the experience, including the addition of external factors that are established elements for understanding the context of access to healthcare in a particular setting; accessibility, availability, acceptability, and quality [50].

## Results

### Participant characteristics

A total of 15 parents participated in the study; four had one child with MLD, 10 had one child living with SMA, and one parent had two children living with SMA. Every child had health insurance through at least one parent’s employer. Nine patients were also double or triple-covered through a public insurance program such as Medicaid. Additional participant characteristics can be found in Table 1. Interviews lasted an average of 29 min.

**Table 1:** Participant characteristics

Characteristic	N	%
Child’s diagnosis		
Spinal muscular atrophy (SMA)	11	73.3
Metachromatic leukodystrophy (MLD)	4	26.7
Relationship to patient (child)		
Mother	14	93.3
Father	1	6.7

	Mean (SD)	Minimum–maximum
Parent age	39 (9.26)	31–67 years old
Child age	6 (5.62)	6 months to 21 years old
Time to diagnosis (months)	13.53 (14.3)	0 (Prior to birth)- 48

	N	%
Region		
South (Maryland, Virginia, Tennessee, Texas)	6	40.0
North (New York, Massachusetts)	3	20.0
Mid-West (Illinois, Michigan, Minnesota)	5	33.3
West (California)	1	6.7
Insurance type		
Employer sponsored	15	100.0
Medicaid	6	40.0
Medicare	1	6.7
Children’s health insurance plan	2	13.3
Medical assistance program (state or county)	2	13.3
Education		
High school diploma	1	6.7
College degree	8	53.3
Graduate degree	6	40.0
Currently employed		
Yes	11	73.3
No	4	36.7

### Major themes

A total of seven major themes were identified: (1) Involvement; (2) Support; (3) Obtaining insurance; (4) Interacting with insurance company representatives; (5) Accessing care through insurance; (6) Financial assistance; (7) Individual factors. Themes are described in detail below with illustrative quotes and additional quotes are reported in Table 2. Sub-themes, including disputes and emotions, provide additional detail to the experience and the model. All quotes are reported using the patients’ disease and state of residence to protect privacy.

**Table 2:** Additional illustrative quotes

Theme	Quote
Obtaining insurance	When we tried to get Medicaid, there are literally hoops that you have to jump through to get them into a Medicaid program, at least in the state of Texas. So, you could either go down to the waiting list that took X number of years – I mean, we’re still on some of these other lists, and she was diagnosed four years ago, so a lot of these lists are, you know, 10, 12, 15 years long, for waiting lists… But you can try the Writer 28, and you have to meet at least two criteria. (MLD, TX)
Obtaining insurance	There was another mom that had just, on the advice of a doctor, had moved from Arkansas to Texas because of the Medicaid benefits. (MLD, TX)
Interacting with insurance company representatives	If you’re trying to manage – you have other children and you work and you’re trying to keep a household and what not, it’s hard to sit on the phone for 30 min waiting for someone to help you, and then, you may get redirected five times. (SMA, CA)
Accessing care	For her, you might need a drug that is proven for cystic fibrosis, but we know for a fact that she has some of the same lung issues, but we may not be able to get the insurance to cover that equipment or that drug because we don't have the background that says, "Oh yeah, they will work for MLD too." (MLD, MN)
Accessing care	When we need things, we've not really had any pushback on them saying, “No, you know you all don't get that.” But I don't feel like we've really asked for crazy things that aren't necessary either. (SMA, TX)
Accessing care: disputes	So we were under the impression that we were being covered, but we weren’t, because our insurance company had a cap we were never made aware of [despite prior inquiries], and therefore, I fought very long to get over $3,000 worth of physical therapy appointments covered by the hospital. (SMA, TX)
Financial assistance	Another device that was not covered is an Eye Gaze communication device was not covered by insurance, and thankfully, the school system provided that for my son while he was in preschool and not physically attending a school yet. So, if we had lived in a county or a district that was unable to do that, we would still not have a way for our son to communicate with us in an understandable way for everyone else. (SMA, MD)
Individual factors	I’m not sure about things like PT and OT, and the reason I don’t know about that right now is because her PT and OT needs currently are covered by our state’s early intervention program, and so, there is no cap for those, so I don’t know. When she reaches the age of three, that may become more of an issue for us. (SMA, TN)
Individual factors: emotional factors	She has a genetic disease– we didn’t know, we didn’t anticipate it, it’s not something that happened because of malpractice or because of negligence or anything. But I certainly want everybody to be able to take care of their kids, their sick kids, as well as we can. (SMA, IL)
Individual factors: emotional factors	It goes back to walking around in somebody else's shoes and trying to figure it out. It is not like we are trying to take advantage of anybody when we have kids with rare genetic illnesses. It is very difficult. (MLD, MN)

### Involvement

Navigating insurance correctly was viewed as a necessity. However, in most responses, individuals did not differentiate between their public and private insurance experience. Individuals expressed confusion related to health insurance documentation, such as benefit descriptions. Some people wanted help finding information, while others did not believe clearer answers existed or that insurance would provide them. Individuals spoke about the iterative process of learning the system and piecing together information over time.

You know, we have talked to a lot of organizations and individuals over the course of her 11 months of life, but I think it has really fallen to us to educate ourselves. We’ve probably talked to 50+ people from advocacy groups, to disability coalition, to lawyers, to case workers, to social workers to, you know, political advocates, and each person has provided us with a little piece of information, but it’s kind of remained up to us to sort of figure out overall how to navigate the system. (SMA, TN)

Parents felt obligated to keep detailed notes, stay actively involved in learning about their child’s disease, and ensuring care needs were met. Even parents who described a more passive approach to seeing what happens as claims moved through the system, still described taking actions, such as sending claims back to providers or pre-writing authorization letters.

### Support

Disease specific organizations, disability organizations, social services agencies, and medical professionals were often seen as a valuable starting point for emotional support and knowledge. Employer benefits managers or members of the leadership team intervened to get benefits on behalf of some of the families. Most individuals spoke about the importance of peer support. This often came from patient communities, including social media.

There was at least one time where I was receiving incorrect information from our insurance company. I was put on hold, because they were trying to figure things out, and I went online, and I said, “Hey, who here has this insurance company and was told that?” and literally 30 seconds later, another mom wrote in to say, “We do. This is what I was told, and this is what you need to tell them.” So, by the time I got off hold, they were like, “Here.” I told them, “Here, this is what it is. No.” I was like telling the insurance company, “this is what it really is.” (SMA, TX)

### Obtaining insurance

All individuals stated their child had never been uninsured. The desire to have immediate coverage for treatments, specifically nusinersen, led one mom to decline anesthesia during childbirth to ensure she could complete the social security paperwork and email her insurance company following her child’s birth. Some participants had difficulty obtaining secondary insurance, which they attributed to state-based eligibility variations or long wait times. MLD parents stated they did not qualify for secondary insurance before they received the diagnosis, but their primary insurance denied coverage for diagnostic testing. Public insurance was described as either a way to improve access to care, such as nursing services, or a way to pay costs associated with private insurance. For example, Medicaid benefits covered copayments associated with employer sponsored plans and in some states, Medicaid paid for a child’s monthly health insurance premiums.

Almost every single state in this country has a waiver that allowed medically disabled children to get on the state Medicaid system, so that they can get access to all of those services, regardless of parental income, and our state does not have that, so that has been incredibly difficult for us, and has been a major barrier in getting her care, you know, nursing, and some of the equipment that is only covered by Medicaid, it’s not covered by private insurance. (SMA, TN)

Employment was a critical factor in obtaining insurance for all participants, but many described the limitations based on the size of their employer or the quality of the plans that were offered. Health insurance was cited as a determining factor for any employment decisions, including one individual who, despite her age, is working to “pay my fair share”. Individuals spoke about fears related to losing a job and repercussions for being “too costly”, despite the legal ramifications of discrimination.

Trying to figure out what insurance we should get was, the hardest part, because I was too scared to call the insurance directly. Like, each of us [both parents] didn’t want to just call them and ask them, because we thought, we might get fired from our jobs, because they would find out how much it costs, because we had heard horror stories… There are laws on the book that protect you, so they can’t fire you, but it happens. Like, all of a sudden, your job just isn’t there anymore, you know? (SMA, VA)

## Interacting with insurance company representatives

Many individuals tried to pre-plan their short and long-term care health needs and work with insurance to see what would be covered in a specified timeframe to anticipate denials or reauthorization periods to limit disruptions in care. Everyone described frustrations related to calling insurance companies, but those whose children were asymptomatic due to current treatment regimes, stated they did not have to do this often. Individuals who had more complex health needs described the time they spent each week on the phone. When individuals contacted the insurance company, they often had to navigate automated systems, first-tier customer service representatives, and multiple people before finding the appropriate person to provide an answer. The frustration was exacerbated by the complications of daily life.

Then that means I’m suctioning his trach [tracheal tube] and having somebody on headphones [from insurance] and helping him to read his guided reading book. You know, that can make it tricky… how time-consuming it is to navigate. (SMA, MD)

Individuals described times when they felt that they received different answers from different people or mentioned inconsistencies in documentation online versus in printed materials. Although a few felt that they understood the benefits, the majority felt that vague language or inconsistencies were purposeful. After an on-going dispute where the family and their hospital believed the insurance company purposefully provided inconsistent information, one mom resorted to threatening to go to the media, after that, the issue was handled in 24 h.

The left hand doesn’t know what the right hand is doing, and that makes it really tricky to navigate, because, you know, it puts more pressure on the parents or the caregiver to do their due diligence, where I feel like it shouldn’t necessarily be all on us to do it. (SMA, TX)

Correctly submitting documentation was described as a team effort between parents and medical providers. Providers submitting claims to the wrong insurer or incorrectly submitting claims using the wrong code could result in claims sitting in limbo for months or denials of routine claims. Certain treatments, referrals, and equipment required reauthorizations or repetitive documentation to continually prove on-going medical necessity.

She doesn't have something that's just going to get better or go away. She’s always going to have it; so I just don't know why we have to keep running through the same circles for the same thing. (SMA, MI)

Some insurance companies provided caseworkers or navigators proactively, while other participants only received caseworkers after requesting one. Caseworker quality was highly variable according to participants but often improved if the caseworker stayed with the family over time. Individuals felt that these individuals had the potential to help navigate the terminology, documentation, or provide cost saving options. People wanted to be treated with a sense of respect. When they did not feel heard or if they were treated like they were trying to “game” the system, it eroded trust, reinforced the need to be vigilant, and to go into “mama bear advocate mode”.

I mean, I hate it [interacting with insurance], but it has to be done, because we can’t afford to not have it be done right, so we just have to continue to keep this documentation of every call and every time and what they said, because I feel like I’m more organized than they are, and I feel that I have to be, because my daughter’s definitely worth it, so this is where we have to be. (SMA, TX)

### Accessing care through insurance

Participants reported the greatest barriers to coverage related to equipment, nursing care, therapy, and out-of-network providers. Those who were aware of tiered-financing schemes indicated their child’s providers were always on the highest tier where they would need to pay the most out-of-pocket. A common sub-theme was disputes when insurance would deny coverage to care which would then force individuals to interact with insurance again. For example, seeking out of network care was discouraged by insurance, but many parents expressed the frustration of not being able to seek disease specific expert care. As one said,

It was recommended that we go to Columbia Children’s Hospital, where they have an SMA clinic, but originally, we were denied coverage there because it was out of network, and basically I said, “Why?” They said I have a pulmonologist center up closer to home… but we would argue there is not an SMA specialist out here. (SMA, NY)

The insurance company’s representative’s comprehension and understanding of the medical situation seemed inadequate to most participants. Due to the lack of knowledge of the disease, parents wanted insurance to try to understand their case history or defer to the medical professionals related to their care needs when making coverage decisions.

She has to be on continuous pulse oximetry monitoring, which we were denied multiple times, until our doctor wrote for oxygen, and she doesn’t need oxygen. In fact, that’s kind of contraindicated in SMA, but the insurance company would not allow us to have a pulse oximeter to monitor her oxygen level in her blood and her adequacy of ventilation, until she was written for oxygen. (SMA, TN)

Providers are responsible for submitting healthcare claims to insurance companies so parents whose children were covered through multiple plans could not always be sure where claims were submitted until they received a statement of benefits, a description of potential out-of-pocket costs, or a bill. Inconsistencies in what parents were ultimately financially responsible for across similar claims necessitated following up on each statement of benefits to avoid unexpected bills. Some aspects of care also required additional steps to avoid out-of-pocket costs, for example, one parent said that every month she must call the durable medical equipment company directly to ensure that the claim will be submitted to insurance, if the company auto-ships the company would not charge insurance and she would be responsible for the bill.

Nurses and therapists provided information, respite help for parents whose children often need around the clock-care, and helped children hit medical milestones. Parents were frustrated by the minimal amount of nursing care and therapy that was covered by insurance, which often seemed inadequate or disrupted care patterns that were showing mobility gains. Multiple people spoke about the annual process of trying to get clarity on coverage allowances, only to be told different information or lose access later.

Some parents felt that their total out-of-pocket costs were reasonable considering the scope and total cost of the care needs. This often came from individuals who had additional coverage, such as employment perks that covered fees or who were on secondary insurance programs. Five individuals were aware of out-of-pocket maximums for the year, four could name what month they met that maximum. There was a split between individuals who just referred to “bills” and those who could recall the exact amount for premiums, deductibles, copays, and coinsurance. Those who had deductibles said they ranged from $1,000 to $7,500 for their family and spoke about how quickly they met them. The cost of equipment, drugs, therapy, and out-of-network services were described as the most expensive components of care. None of the individuals received a denial for nusinersen, however, many were concerned about the waiting period before the cost was covered or before they received the approval for the drug. These types of services often had cost-sharing mechanisms, but the patient’s portion was still quite large. As one participant said, “A 10% copay on a $150,000 per year bill is prohibitive for most people”. (SMA, TN).

A few individuals spoke about shifting formularies, gatekeeping requirements, networked providers, and benefits that sometimes changed how the individual could access care. Sometimes these changes would be related to eligibility shifts, such as a provider who was included in Early Intervention, but out-of-network when the child aged-out. Many people discussed the concern about the health consequences of the delays or denials of the insurance company, especially the potential for worst health outcomes or lost opportunities, such as clinical trial participation. Many expressed that if a doctor indicated that something was medically necessary, families should have an affordable way to access it. For example, one participant said,

It shouldn’t be about, “Oh, sorry, you can’t have this because it’s too expensive.” Well, but that’s what the patient needs. So, if that’s what the patient needs, find a way to make it more affordable so that they can have it. (SMA, CA)

Participants had different expectations about what should and should not be covered by health insurance. Some individuals were narrowly focused on specific medical costs, while others looked at the full paradigm of care and supportive technologies, such as powerchairs and adaptive beds, that impacted overall health outcomes.

Now, I am trying to get some equipment for her mattress and I am just trying to get it paid for with the proper paperwork signed and everything… I feel if I did the financial analysis on how much it costs to take care of a wound, they would certainly rather pay for the mattress, it is 300 bucks instead of the $3,000 that it is going to cost if I have to put her in the hospital time and time again. (MLD, MN)

### Financial assistance

Insurance was described as one piece in the larger financial structure. Almost every SMA patient received nusinersen through the clinical trial or the drug company’s copay assistance program. Many utilized equipment shares, local charities, small grants, or personal fundraisers to meet additional needs. If an individual did not access other financial assistance programs they indicated either none were available, they were saving it for a future need, they did not have time to complete the applications, or they believed others had greater need. Responsibility fell to parents to identify the source of funds and decide the timing of the of the application.

And I really think that when you look at someone's care needs you know insurance is one part of it but it's really like trying to understand all of the benefits including insurance that they are entitled to and how all of those pieces need to work together. (SMA, MN)

### Individual factors

The child’s health was a sub-theme within individual factors. Some parents reported that after their child took nusinersen they no longer had on-going care needs. In these cases, parents were less likely to have on-going disputes with insurance companies and were more focused on the initial struggle to access the drug.

When asked if individuals were satisfied with their insurance, many said yes, despite describing challenging experiences. One respondent stated, “That’s kind of a trick question.” A few reflected on the importance of taking care of each other. Individuals also looked at their own privilege related to education, support, and the severity of their child’s condition when reflecting on their experience.

A family that the parents are working two jobs and they are barely able to look over their bills and they just have time to pay them, if they don’t have time to scrupulously look through what is actually being billed, there’s been times where we’ve had more than $1,000 of a bill for something that was billed incorrectly, and then that turns out to be kind of an insurance nightmare (SMA, MD)

People spoke about how the coverage they could access would impact their life decisions, such as having more children. Many expressed that things were not fully in their control and they only had so much bandwidth to continually fight. The political climate and fears of losing protections for preexisting conditions weighed heavily on many. A few people spoke directly about their own mental health.

It goes back to walking around in somebody else's shoes and trying to figure it out. It is not like we are trying to take advantage of anybody when we have kids with rare genetic illnesses. It is very difficult. (MLD, MN)

## Model

**Fig. 1:**
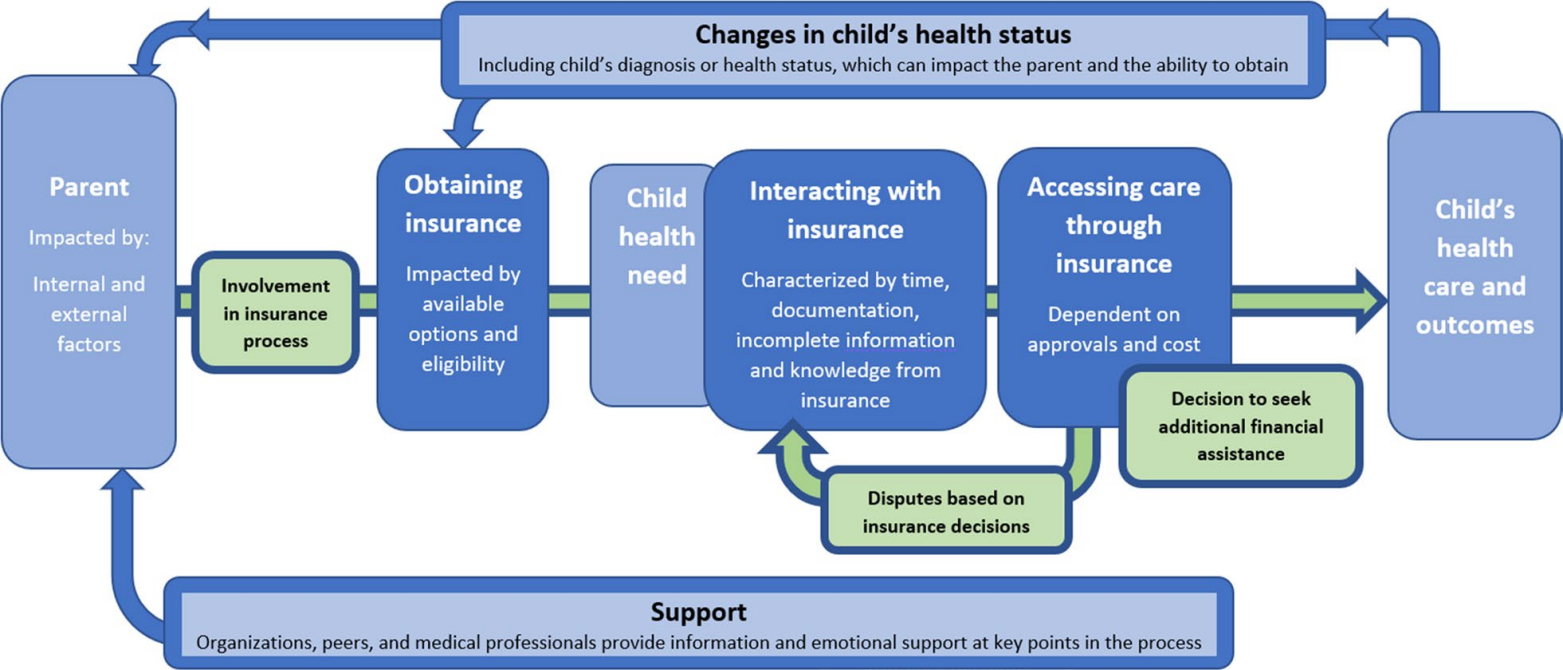
An explanatory model of health insurance experiences of parents of children with rare diseases in the US

An explanatory model of health insurance experiences of parents of children with rare diseases in the U.S. (Fig. 1) was developed using themes generated by analysis of the interviews and previously established external factors that affect healthcare access. Although the model was created based on the responses of parents, it could also apply to any primary caregiver of a rare disease child. The model demonstrates how the parent needs to be involved throughout the process and the parent’s focus on their child’s long-term health outcome. Characteristics related to the parent and child are displayed in light blue. Areas in green represent the actions taken by the parent to navigate the insurance system to ultimately access care on behalf of their child to influence aspects of care and overall health outcomes. The parent’s ability to navigate the system was impacted by **individual factors** such as demographics, location, employment, health literacy, and emotional factors. Emotional factors such as uncertainty, urgency, and responsibility impacted the level of “hands-on” involvement parents exhibited while navigating insurance. Established external factors of accessibility, availability, acceptability, and quality [50] impact an individual’s health quality and the opportunity to obtain insurance. Emotional and knowledge **support** was provided by external forces throughout the process, but it was still up to the parent to coordinate care.

The domains in the model identified in dark blue, obtaining, interacting, and accessing, refer to direct parental interactions with health insurance companies. **Obtaining** insurance can be complicated by eligibility constraints and options within exchanges or employers. **Interacting** with the insurance company required time and complex documentation and was categorized by frustration in the redundancy of needed reauthorizations, incomplete information, and sub-par knowledge by the company representatives. **Accessing** care through insurance describes the approved coverage of healthcare services an individual receives through health insurance. However, disputes may arise if services are denied or are too costly for patients. Additional financial **assistance** may be sought outside of the health insurance system, such as drug co-pay or personal fundraising programs. The result of this additional access or delays in care can have a positive or negative impact on the child’s health outcomes. These changes, either improvements, such as access to an orphan drug or a new diagnosis, or medical setbacks, such as an additional hospitalization, can result in eligibility changes or the approach a parent may take when interacting with the system.

## Discussion

Parents in this study reported a need to meticulously track time-consuming interactions with insurance company representatives, and often felt that they are missing key information about coverage allowances and what care was covered for their child under their insurance policy. A single health insurance policy was rarely enough to cover the cost of all healthcare needs for their child, which resulted in parents viewing access as a web of different policies and social service supports.

In this study, parents of children with both SMA and MLD had to interact extensively with insurers to gain access to services and products seen as medical necessities, which is consistent with previous findings [15, 16, 28]. None of the SMA parents spoke about delays in diagnosis and some SMA parents had learned of their child’s diagnosis in the prenatal period. Early establishment of a diagnosis made initial interactions with insurance companies somewhat easier for parents of children with SMA compared to parents of children with MLD, which often takes more time to diagnose. Parents, with recommendations from medical professionals, felt that they were looking at long-term needs and outcomes, but parents felt that insurers were more focused on more immediate utilization controls and reducing access to expensive drugs and services. Both MLD and SMA parents felt that they had to educate insurance company representatives about their child’s disease and justify care needs. However, it appeared that insurance companies had fewer established guidelines for MLD, which necessitated more intervention from allied health professionals or employers working with parents of a child with MLD to facilitate access care when compared to parents of a child with SMA.Previous studies speak to the lack of knowledge among medical professionals for rare diseases [4, 7], but lack of knowledge within insurance companies has not been described. The lack of knowledge parents perceived by insurance representatives led to greater frustrations when insurers did not consider the medical benefits and long-term cost savings of access to equipment, therapy, and diagnostics. This is consistent with studies that have indicated barriers to diagnosis and diagnostic testing for rare diseases [7, 51]. Parents in this study expressed frustration that insurance representatives did not understand the importance of seeing disease experts or maintaining treatment schedules set-up by these specialists.

The use of patient navigators from within insurance companies to help people navigate the clinical aspects of the healthcare system have not systematically been described in the literature. Navigators are often social workers or medical professionals who can help expand coverage, provide referrals, provide support, help navigate the system, and provide care coordination [52–54]. There are a number of studies dedicated to their use for people with chronic conditions, the majority of which focus on cancer [52, 55]. One mother stated that hospital navigators “can help you while you are inpatient and actively in treatment, but we don't have that sort of thing for just the after mess [on-going medical needs] with leukodystrophy.” These programs show promise for increasing access and addressing health disparities in chronic conditions and may benefit rare disease parents [54, 55].

Many inequities exist in the U.S. healthcare system and, although the extent has not been fully determine, are likely to negatively affect individuals living with genetic or rare diseases similarly to other populations of patients [56]. Rare diseases are not limited to certain sociodemographic populations and social determinants of health such as income, race, community, access to services, social supports, and education are likely to affect the timeliness and quality of care patients receive [57]. Investing in a just healthcare system that addresses structural and systemic barriers will be beneficial to all [58–60].

There is a growing dialogue on the need to address the negative impact of the high costs associated with expensive treatments [30, 61]. In rare diseases, there is additional criticism related to the high price tag of orphan drugs [34, 62–64]. Study participants were not always in agreement related to which healthcare costs should be covered by the system, especially for supportive technologies or medical devices that individuals believed could prevent future healthcare needs. Inequities in the U.S. system, growing criticisms around the cost of orphan drugs, and additional calls for addressing unmet need will require additional healthcare debates and new policies [30, 59, 61–65].

### Policy considerations

Individuals in the U.S. managing chronic conditions, such as musculoskeletal problems or lung conditions, may face access issues that can lead to unmet health care needs [66, 67]. The concerns with access and high costs of care are similar between those with chronic conditions and with rare diseases. Additionally, parents of children with other common chronic diseases in the U.S face challenges similar to parents of children with a rare disease related to the need to coordinate healthcare while navigating evolving healthcare needs [68]. Although caregivers or patients with chronic conditions are encouraged to learn about their disease and its treatment so that they can self-manage their disease between visits with physicians, patients with rare diseases or their caregivers must often be the expert on all nuances of the disease because primary care providers are not always knowledgeable enough about the condition to provide key aspects of coordination [11, 15, 16]. Difficulties in determining a diagnosis are less common for children with other chronic diseases, an issue that can complicate gaining access to targeted healthcare programs, such as Medicaid and Medicare for parents of children with a rare disease [7, 30]. Investing in programs that could lead to more timely diagnosis, including access to genetic testing could benefit patients [13, 51]. Additionally, expanding access to Medicaid and the resources to limit the time necessary to review an application could result in additional avenues to obtain insurance.

Insurers are unlikely to be knowledgeable about all rare diseases, but if insurers were to provide a clearer framework for assessing medical needs or employed specialists who are trained to work with complex care cases in a respectful way it would improve the parent experience. Some insurers utilize this type of specialist and patient navigators for chronic care patients have helped to improve patient outcomes, address disparities, and reduce the cost of care for patients [54, 55, 69, 70]. However, little, has been done to evaluate the effectiveness of patient navigators for improving outcomes and costs among rare disease patients. Rare disease specific navigators can also more appropriately provide information about the landscape for additional financial assistance opportunities.

This study adds to the literature that documents the importance of prior authorizations being evidence based, timely, and incorporate the expertise of medical professionals familiar with the disease [14, 16, 71]. Policies should focus on increasing the transparency of the preauthorization and claims process and establishing time limits for processing coverage decisions [5, 15, 71]. Universal authorizations for on-going needs or specialists would decrease the burden on families and medical providers. Individuals with compelling needs to see a disease specialist should have an opportunity to seek waivers from out-of-network care cost restraints or to seek expert opinions in a cost-effective way, which may be leveraged through the increased use of telemedicine. More research into the natural history of rare diseases and establishing medically recognized treatment guidelines would improve evidence-based care for patients, especially if insurance was required to provide coverage for the services necessary to follow those guidelines.

Rare disease patients and families across the globe and different healthcare systems have expressed challenges finding care and experts knowledgeable in their disease [6, 7, 16, 61]. However, some countries have made more significant attempts to address these problems by establishing rare disease centers of excellence and national rare disease plans [72, 73]. There are some similar initiatives through the NIH and patient advocacy organizations at the state and federal level that could be supported to further facilitate access to care [1, 74]. Additional awareness and research related to the needs for families living with rare diseases can further highlight additional policy considerations. Additional policy recommendations are explored in Table 3.

**Table 3:** Major themes and policy recommendations

Major themes	Primary obstacle/barrier	Policy recommendation
Obtaining insurance	Insurance eligibility differences across states and wait lists to obtain public insurance can limit access to certain types of care Life decisions such as employment and geographic location are tied to healthcare needs	Consistent mechanisms for patients living with rare diseases to enroll in insurance programs Universal coverage programs that are not tied to employment Additional resources in Medicaid to reduce enrollment wait times Programs to help patients access diagnostics, including genetic testing
Interacting with insurance company representatives	Difficulty getting clear and consistent answers related to coverage Time intensive and redundant process to cover on-going care needs Lack of knowledge amongst insurance company representatives about the medical condition and care needs	Insurance staff trained in dealing with rare diseases A better framework to assess rare medical needs Assigned caseworkers or patient navigators within insurance and a direct way for parents to contact their representative to increase consistency Clear and transparent documentation related to coverage benefits Increased transparency in the claims and prior authorization processes to decrease the time and understand the status Time limits for coverage decisions Universal authorizations for on-going needs to decrease redundancy
Accessing care through insurance	Changes from year to year resulted in different out-of-pocket costs and an ability to plan for other healthcare assistance If coverage was denied, parents were forced to interact with insurance again to dispute the claim Some aspects of care were seen as medically necessary by providers, but were not covered under insurance Cost-sharing mechanisms, even if modest, could be prohibitive	Consistency of coverage across plan years and clarity around changes Published fee schedules and costs Waivers for out-of-network care so individuals can access diseFinancial assistance or caps on total out-of-pocket costs ase experts Approvals for telemedicine that are not subjected to out of network care restraints
Financial assistance	Additional financial assistance was often necessary to cover healthcare needs From the parental viewpoint, insurance and other assistance were an interconnected web to cover needed care Some programs were dependent on age or geography	Centralized location for information about other financial assistance programs Rare disease specific navigators
Involvement in insurance	Parents were responsible for learning the system and available options Health literacy and overall comfort interacting the system could impact mental health and stress Parents felt they needed to devote a lot of time to understanding the system, especially to prevent a health event or setback	Trained rare disease patient navigators or centralized information sources Increased awareness for the challenges facing rare disease families Additional research related to the experience of navigating the healthcare system and strategies to facilitate better care

## Limitations

MLD parent recruitment was more challenging based on the estimated prevalence of the condition and the severity of the condition. Fewer parents of children with MLD participated in the study, which may have limited the understanding of parents’ experiences related to a disease without an FDA approved treatment. There may have been selection bias as individuals self-selected to participate in the study. Participants who enrolled may have been more comfortable talking about insurance than those who decided not to participate. Those who did not enroll may have had more time constraints or stress that prevented their participation. Additionally, individuals who were not connected to the patient organizations would not have seen the messages, which might mean our results over-report the importance of organizational support. It is possible that later enrollees of the study were encouraged by earlier participants to enroll in the study. It is therefore possible that these participants had similar experiences and viewpoints as others who had already participated in the study, further limiting the diversity of experiences included.Study participants were more likely to be female, live in the Southern U.S., and have at least a college degree. It was not surprising that the majority of respondents were female because mothers are most often the primary caregiver for children with rare diseases in the U.S. and are often responsible for coordinating care for their child [75]. Although two participants said they were not sure what their monthly health insurance premium was because it was automatically paid through their spouse’s paycheck, no other gaps in knowledge about the child’s insurance experience were apparent. U.S. Medicaid programs in southern states tend to have stricter eligibility criteria and fewer benefits, no regional pattern to the themes that emerged was noted. The sample also included individuals with higher educational attainment than the majority of Americans, these individuals may have higher digital literacy or a higher comfort level with research which resulted in them being more willing to participate in the study. Families with lower educational attainment may experience even greater challenges interfacing with health insurers and obtaining recommended care (66). Further study is needed to determine whether there may be additional gaps in care coordination and health literacy that could be addressed through health care policy.

None of the participants in the study were uninsured or exclusively on public insurance, which could limit the applicability of the model to those populations. Insurance status was self-reported and cannot be validated but is likely accurate due to the level of involvement of most parents with insurance companies and their representatives. Participants in this study discussed equipment and therapy as these are key features in diseases with mobility issues, but other rare diseases are likely to have somewhat different needs. Finally, although we reached data saturation in our interviews, it is possible that if more interviews were conducted across a larger sample of the population additional insights and themes might emerge.

## Conclusion

Participants viewed insurance as just one component of a larger puzzle that allows them to access necessary care for their child. Insurance companies are often ill-equipped to provide clear consistent answers on a disease they know little about, forcing parents to meticulously track insurance benefits and interactions to balance medical needs and financial stability. The complexity of the U.S. insurance system requires parents to enroll in multiple plans to maximize coverage, an option that is not available for all families. Individuals are often grateful for a supportive network of peers and providers to identify program eligibility for additional assistance, but the final responsibility falls to them. There are policy initiatives that could impact payment and delivery systems that could greatly improve patient experience and outcomes. Incorporating the caregiver and patient perspective is critical in any reform effort. Additional studies are needed to understand the full scope of barriers to care and policies that can facilitate better care access for families living with rare diseases.

## Data Availability

The datasets generated and analyzed during the current study are not publicly available due to participant privacy concerns but are available from the corresponding author on reasonable request.

## Abbreviations

EI: Early intervention
FDA: Food and drug administration
MLD: Metachromatic leukodystrophy
OD: Orphan drugs
ODA: Orphan drug act
SMA: Spinal muscular atrophy
NIH: The National Institutes of Health

## References

[1] Genetic and Rare Diseases Information Center (GARD), National Center for Advancing Translational Sciences. Genetic and Rare Diseases Information Center (GARD) – an NCATS Program [Internet]. NIH.gov. [cited 2017 Jan 13]. https://rarediseases.info.nih.gov/

[2] Orphan Drug Act [Internet]. Federal Food, Drug, and Cosmetic Act, 97–414 1983. https://www.ecfr.gov/cgi-bin/text-idx?c=ecfr&SID=51cf70689d51f0ea4147c0a8ac649321&rgn=div5&view=text&node=21:5.0.1.1.6&idno=21

[3] National Organization for Rare Disorders (NORD) [Internet]. NORD (National Organization for Rare Disorders). [cited 2017 Apr 19]. https://rarediseases.org/

[4] Babac A, Frank M, Pauer F, Litzkendorf S, Rosenfeldt D, Lührs V (2018). Telephone health services in the field of rare diseases: a qualitative interview study examining the needs of patients, relatives, and health care professionals in Germany. BMC Health Serv Res..

[5] Pelentsov LJ, Fielder AL, Laws TA, Esterman AJ (2016). Development of the parental needs scale for rare diseases: a tool for measuring the supportive care needs of parents caring for a child with a rare disease. J Multidiscip Healthc.

[6] Pelentsov LJ, Fielder AL, Laws TA, Esterman AJ (2016). The supportive care needs of parents with a child with a rare disease: results of an online survey. BMC Fam Pract.

[7] Kole A, Faurisson F (2010). Rare diseases social epidemiology: analysis of inequalities. Adv Exp Med Biol.

[8] Report of The National Commission on Orphan Diseases [Internet]. 1989 Feb [cited 2017 Mar 31]. https://rarediseases.info.nih.gov/files/report_of_the_national_commission_on_orphan_diseases_february_1989.pdf

[9] Groft SC, de la Paz MP (2010). Rare diseases - avoiding misperceptions and establishing realities: the need for reliable epidemiological data. Adv Exp Med Biol.

[10] Somanadhan S, Larkin PJ (2016). Parents’ experiences of living with, and caring for children, adolescents and young adults with Mucopolysaccharidosis (MPS). Orphanet J Rare Dis..

[11] Palacios-Ceña D, Famoso-Pérez P, Salom-Moreno J, Carrasco-Garrido P, Pérez-Corrales J, Paras-Bravo P, et al. “Living an Obstacle Course”: A Qualitative Study Examining the Experiences of Caregivers of Children with Rett Syndrome. Int J Environ Res Public Health. 2018 25;16(1). 10.3390/ijerph16010041 PMC6338949 30585176

[12] Lagae L, Irwin J, Gibson E, Battersby A (2019). Caregiver impact and health service use in high and low severity Dravet syndrome: a multinational cohort study. Seizure.

[13] Gallo AM, Hadley EK, Angst DB, Knafl KA, Smith CAM (2008). Parents’ concerns about issues related to their children’s genetic conditions. J Spec Pediatr Nurs.

[14] Curtis K, Foster K, Mitchell R, Van C (2016). Models of care delivery for families of critically ill children: an integrative review of international literature. J Pediatr Nurs.

[15] Baumbusch J, Mayer S, Sloan-Yip I. Alone in a crowd? Parents of children with rare diseases’ experiences of navigating the healthcare system. J Genet Couns. 2018 Aug 21; 10.1007/s10897-018-0294-9 30128673

[16] Currie G, Szabo J (2019). “It is like a jungle gym, and everything is under construction”: the parent’s perspective of caring for a child with a rare disease. Child Care Health Dev.

[17] American Academy of Pediatrics Council on Children with Disabilities. Care coordination in the medical home: integrating health and related systems of care for children with special health care needs. Pediatrics. 2005;116(5):1238–44. 10.1542/peds.2005-2070 16264016

[18] Aday LA, Andersen R (1974). A framework for the study of access to medical care. Health Serv Res.

[19] Levesque J-F, Harris MF, Russell G (2013). Patient-centred access to health care: conceptualising access at the interface of health systems and populations. Int J Equity Health.

[20] Andrulis DP (1998). Access to care is the centerpiece in the elimination of socioeconomic disparities in health. Ann Intern Med.

[21] Committee on Geographic Variation in Health Care Spending and Promotion of High-Value Care, Board on Health Care Services, Institute of Medicine. Variation in Health Care Spending: Target Decision Making, Not Geography [Internet]. Newhouse JP, Garber AM, Graham RP, McCoy MA, Mancher M, Kibria A, editors. Washington (DC): National Academies Press (US); 2013. http://www.ncbi.nlm.nih.gov/books/NBK201647/24851301

[22] Health Insurance Coverage of the Total Population [Internet]. The Henry J. Kaiser Family Foundation. 2017 [cited 2017 Sep 22]. http://www.kff.org/other/state-indicator/total-population/

[23] Centers for Medicare & Medicaid Services. CMS Fast Facts Overview [Internet]. CMS.gov. 2017 [cited 2017 Sep 22]. https://www.cms.gov/Research-Statistics-Data-and-Systems/Statistics-Trends-and-Reports/CMS-Fast-Facts/index.html

[24] September 2020 Medicaid & CHIP Enrollment Data Highlights | Medicaid [Internet]. [cited 2021 Mar 10]. https://www.medicaid.gov/medicaid/program-information/medicaid-and-chip-enrollment-data/report-highlights/index.html

[25] Mays GP, Claxton G, White J. Managed care rebound? Recent changes in health plans’ cost containment strategies. Health Aff Proj Hope. 2004;Suppl Web Exclusives:W4–427–36. 10.1377/hlthaff.w4.427 15451964

[26] Obama B (2016). United States health care reform: progress to date and next steps. JAMA.

[27] Orszag PR (2016). US health care reform: cost containment and improvement in quality. JAMA.

[28] Pelentsov LJ, Fielder AL, Esterman AJ (2016). The Supportive Care Needs of Parents With a Child With a Rare Disease: A Qualitative Descriptive Study. J Pediatr Nurs.

[29] Kuester MK, Jackson EA, Runyan BM, Pezalla EJ, Nussbaum SR (2018). The effect of a pediatric rare disease on subscriber retention rates for commercial health insurers in the United States. J Manag Care Spec Pharm.

[30] Burden of Rare Disease Study [Internet]. EveryLife Foundation for Rare Diseases. [cited 2021 Mar 9]. https://everylifefoundation.org/burden-study/

[31] U.S. Food & Drug Administration. Search Orphan Drug Designations and Approvals [Internet]. [cited 2017 Mar 31]. https://www.accessdata.fda.gov/scripts/opdlisting/oopd/index.cfm

[32] Alhawwashi S, Seoane-Vazquez E, Eguale T, Rodriguez-Monguio R (2016). Prices of drugs for chronic use with orphan designation in the United States (1983–2014). Value Health.

[33] American Health Insurance Plans (AHIP). Orphan Drug Utilization and Pricing Patterns (2012–2014) [Internet]. Washington, DC; 2016 Oct [cited 2017 Sep 22]. https://www.ahip.org/wp-content/uploads/2016/10/OrphanDrug_DataBrief_10.21.16.pdf

[34] Simoens S (2011). Pricing and reimbursement of orphan drugs: the need for more transparency. Orphanet J Rare Dis.

[35] Divino V, DeKoven M, Kleinrock M, Wade RL, Kaura S (2016). Orphan drug expenditures in the United States: a historical and prospective analysis, 2007–18. Health Aff Proj Hope.

[36] Robinson SW, Brantley K, Liow C, Teagarden JR (2014). An early examination of access to select orphan drugs treating rare diseases in health insurance exchange plans. J Manag Care Spec Pharm.

[37] Finkel RS, Mercuri E, Darras BT, Connolly AM, Kuntz NL, Kirschner J (2017). Nusinersen versus sham control in infantile-onset spinal muscular atrophy. N Engl J Med..

[38] Genetics Home Reference, U.S. National Library of Medicine. Spinal muscular atrophy [Internet]. Genetics Home Reference. [cited 2019 Feb 7]. https://ghr.nlm.nih.gov/condition/spinal-muscular-atrophy

[39] Genetics Home Reference, U.S. National Library of Medicine. Metachromatic leukodystrophy [Internet]. Genetics Home Reference. [cited 2019 Feb 7]. https://ghr.nlm.nih.gov/condition/metachromatic-leukodystrophy

[40] Landfeldt E, Edström J, Sejersen T, Tulinius M, Lochmüller H, Kirschner J (2019). Quality of life of patients with spinal muscular atrophy: a systematic review. Eur J Paediatr Neurol EJPN Off J Eur Paediatr Neurol Soc.

[41] Eichler FS, Cox TM, Crombez E, Dali CÍ, Kohlschütter A (2016). Metachromatic leukodystrophy: an assessment of disease burden. J Child Neurol.

[42] Office of the Commissioner. Press Announcements - FDA approves first drug for spinal muscular atrophy. 2016 Dec 23 [cited 2019 Feb 7]. https://www.fda.gov/newsevents/newsroom/pressannouncements/ucm534611.htm

[43] Metachromatic Leukodystrophy [Internet]. National Organization for Rare Disorders; 2003 [cited 2019 Mar 4]. (Rare Disease Database). https://rarediseases.org/rare-diseases/metachromatic-leukodystrophy/

[44] Glaser BG, Strauss AL (1967). The discovery of grounded theory: strategies for qualitative research.

[45] Birks M, Mills J. Grounded Theory: A Practical Guide. SAGE; 2015. 209 p.

[46] Chun Tie Y, Birks M, Francis K. Grounded theory research: A design framework for novice researchers. SAGE Open Med [Internet]. 2019 Jan 2 [cited 2021 Mar 3];7. https://www.ncbi.nlm.nih.gov/pmc/articles/PMC6318722/10.1177/2050312118822927 PMC6318722 30637106

[47] Faugier J, Sargeant M (1997). Sampling hard to reach populations. J Adv Nurs.

[48] Strauss AL, Corbin JM (1998). Basics of qualitative research : techniques and procedures for developing grounded theory.

[49] How Many Interviews Are Enough?: An Experiment with Data Saturation and Variability - Greg Guest, Arwen Bunce, Laura Johnson, 2006 [Internet]. [cited 2019 Apr 30]. https://journals.sagepub.com/doi/abs/10.1177/1525822X05279903

[50] UN Committee on Economic, Social and Cultural Rights (CESCR). General Comment No. 14: The Right to the Highest Attainable Standard of Health (Art. 12 of the Covenant) [Internet]. 2000 Aug [cited 2020 Jul 14]. https://www.refworld.org/docid/4538838d0.html

[51] Reuter CM, Kohler JN, Bonner D, Zastrow D, Fernandez L, Dries A (2019). Yield of whole exome sequencing in undiagnosed patients facing insurance coverage barriers to genetic testing. J Genet Couns.

[52] Wells KJ, Valverde P, Ustjanauskas AE, Calhoun EA, Risendal BC (2018). What are patient navigators doing, for whom, and where? A national survey evaluating the types of services provided by patient navigators. Patient Educ Couns.

[53] Darnell JS (2013). Navigators and assisters: two case management roles for social workers in the Affordable Care Act. Health Soc Work.

[54] Natale-Pereira A, Enard KR, Nevarez L, Jones LA (2011). The role of patient navigators in eliminating health disparities. Cancer.

[55] McBrien KA, Ivers N, Barnieh L, Bailey JJ, Lorenzetti DL, Nicholas D (2018). Patient navigators for people with chronic disease: A systematic review. PloS One..

[56] Fraiman YS, Wojcik MH (2021). The influence of social determinants of health on the genetic diagnostic odyssey: who remains undiagnosed, why, and to what effect?. Pediatr Res.

[57] Marmot M, Allen JJ (2014). Social determinants of health equity. Am J Public Health.

[58] Alcaraz KI, Wiedt TL, Daniels EC, Yabroff KR, Guerra CE, Wender RC (2020). Understanding and addressing social determinants to advance cancer health equity in the United States: A blueprint for practice, research, and policy. CA Cancer J Clin.

[59] Daniels N (2006). Equity and population health: toward a broader bioethics agenda. Hastings Cent Rep.

[60] Daniels N, Kennedy BP, Kawachi I (1999). Why justice is good for our health: the social determinants of health inequalities. Daedalus.

[61] Angelis A, Tordrup D, Kanavos P (2015). Socio-economic burden of rare diseases: A systematic review of cost of illness evidence. Health Policy Amst Neth.

[62] Kesselheim AS, Myers JA, Solomon DH, Winkelmayer WC, Levin R, Avorn J. The Prevalence and Cost of Unapproved Uses of Top-Selling Orphan Drugs. PLoS ONE [Internet]. 2012 Feb 21;7(2). http://www.ncbi.nlm.nih.gov/pmc/articles/PMC3283698/10.1371/journal.pone.0031894 PMC3283698 22363762

[63] Hyde R, Dobrovolny D (2010). Orphan drug pricing and payer management in the United States: Are we approaching the tipping point?. Am Health Drug Benefits.

[64] Hemphill TA (2010). Extraordinary pricing of orphan drugs: is it a socially responsible strategy for the US pharmaceutical industry?. J Bus Ethics..

[65] Carrera PM, Kantarjian HM, Blinder VS (2018). The financial burden and distress of patients with cancer: understanding and stepping-up action on the financial toxicity of cancer treatment. CA Cancer J Clin.

[66] Callahan ST, Cooper WO (2006). Access to health care for young adults with disabling chronic conditions. Arch Pediatr Adolesc Med.

[67] Wilper AP, Woolhandler S, Lasser KE, McCormick D, Bor DH, Himmelstein DU (2008). A national study of chronic disease prevalence and access to care in uninsured US adults. Ann Intern Med..

[68] Bodenheimer T (2008). Coordinating care–a perilous journey through the health care system. N Engl J Med.

[69] Gunn C, Battaglia TA, Parker VA, Clark JA, Paskett ED, Calhoun E (2017). What makes patient navigation most effective: defining useful tasks and networks. J Health Care Poor Underserved.

[70] Rocque GB, Pisu M, Jackson BE, Kvale EA, Demark-Wahnefried W, Martin MY (2017). Resource use and medicare costs during lay navigation for geriatric patients with cancer. JAMA Oncol.

[71] ACMG Board of Directors (2018). Insuring patient access and affordability for treatments for rare and ultrarare diseases: a policy statement of the American College of Medical Genetics and Genomics. Genet Med Off J Am Coll Med Genet.

[72] Khosla N, Valdez R (2018). A compilation of national plans, policies and government actions for rare diseases in 23 countries. Intractable Rare Dis Res.

[73] Castro R, Senecat J, de Chalendar M, Vajda I, Dan D, Boncz B (2017). Bridging the Gap between Health and Social Care for Rare Diseases: Key Issues and Innovative Solutions. Adv Exp Med Biol.

[74] Project RDAC - Overview [Internet]. NORD (National Organization for Rare Disorders). [cited 2021 Mar 10]. https://rarediseases.org/rdac-overview/

[75] Rare Disease Caregiving | The National Alliance for Caregiving [Internet]. [cited 2021 Mar 7]. https://www.caregiving.org/rare/

